# Long-term survival after initial hospital admission for peripheral arterial disease in the lower extremities

**DOI:** 10.1186/1471-2261-9-43

**Published:** 2009-08-28

**Authors:** I Vaartjes, GJ de Borst, JB Reitsma, A de Bruin, FL Moll, DE Grobbee, ML Bots

**Affiliations:** 1 Julius Center for Health Sciences and Primary Care, University Medical Center Utrecht, Utrecht, the Netherlands; 2 Department of Vascular Surgery, University Medical Center Utrecht, Utrecht, the Netherlands; 3 Department of Clinical Epidemiology and Biostatistics, Academic Medical Center, Amsterdam, the Netherlands; 4 Statistics Netherlands, The Hague, the Netherlands

## Abstract

**Background:**

As the population ages, peripheral arterial disease (PAD) in the lower extremities will become a larger public health problem. Awareness in patients as well clinicians of the high risk of morbidity and mortality is important but seems currently low. Insights in absolute mortality risks following admission for PAD in the lower extremities can be useful to improve awareness as they are easy to interpret.

**Methods:**

A nationwide cohort of 4,158 patients with an initial admission for PAD in the lower extremities was identified through linkage of the national hospital and population register in 1997 and 2000.

**Results:**

Over 60% of 4,158 patients were men. 28 days, 1 year and 5 year mortality risk were 2.4%, 10.3% and 31.0% for men and 3.5%, 10.4% and 27.4% for women. Coronary heart disease and stroke were frequent cause of death. Five years mortality risk was higher for men compared to women (HR 1.36, 95% CI 1.21–1.53).

**Conclusion:**

In conclusion, our findings demonstrate that, 5 year mortality risk is high, especially in men and comparable to that of patients admitted for acute myocardial infarction or ischemic stroke. Though, in general population the awareness of the severity of PAD in the lower extremities is significantly lower than that for any other cardiovascular disease and it seems that cardiovascular risk factor management for prevention in PAD patients is very modest.

## Background

Peripheral arterial disease (PAD) is a manifestation of atherosclerosis, affecting an estimated 27 million people in Europe and North America [1]. Intermittent claudication (IC) is the most common symptom of PAD in the lower extremities. IC has a severe impact on various aspects of quality of life [2,3], on the ability to continue to work and approximately 15% to 20% of the patients with IC develop critical limb ischemia (CLI) [4,5]. Furthermore, patient with IC have an increased mortality risk compared to the general population [6,7].

Previous studies reported that individuals with PAD have a 20–60% increased risk for myocardial infarction, a two- to six-fold increased risk of cardiovascular death, and a 40% increased risk of stroke compared to those without PAD [5,8,9]. In spite of many publications reporting an increased risk of morbidity and mortality, patients with PAD lack knowledge regarding the risk of heart attack, stroke and total mortality [10]. Furthermore, the use of secondary prevention treatment in these patients have shown to be modest at best [11].

Although PAD in the lower extremities is rare among young individuals its prevalence increases dramatically with age [7,12]. As the population ages, PAD in the lower extremities will become a greater public health problem [7,12], leading to an increasing necessity for primary and secondary prevention. Awareness in patients as well clinicians of the high risk of morbidity and mortality is therefore important. Most available literature, including institutional and population-based studies, present overall mortality risks for patients with PAD in the lower extremities [13-17] or use relative risks or odds ratio's when reporting mortality [8,11,16]. However, absolute mortality risks stratified by age and gender may be more useful as absolute risks are easier to interpret and they provide a clearer presentation of the size of the burden of disease in these patients. We set out to study absolute mortality risk stratified by age and gender after first hospital admission for PAD in the lower extremities.

## Methods

### Registries and linkage procedure

To construct a cohort of patients admitted for the first time because of PAD in the lower extremities, information from the national Hospital Discharge Registry (HDR) and the Dutch Population Registry (PR) were linked. Information on cause of death was derived from the cause of death registry of Statistics Netherlands. The registries and linkage procedures have been described in detail previously for a cohort of acute myocardial infarction patients [18] and stroke [19]. In brief, the HDR is a nationwide database on admissions, not persons. For each hospital admission a new record is created in the HDR. Following individuals over time based on HDR-information alone is troublesome due to difficulties in identification of different admissions from the same person in time and admissions for the same condition at a different hospital (due to referral or to address changes). Yet, linkage (linkage variables date of birth, gender and 4 digits of postal code) with the PR may overcome these issues.

For the present study, cohorts were extracted from 1997 and 2000 because of pragmatic reasons at the time of the initiation of the project in 2001. The total population of the Netherlands in 1997 and 2000 was 15,567,107 (men 7,696,803, women 7,870,304) and 15,863,950 (men: 7,846,317, women: 8,017, 633), respectively.

### Definition

PAD in the lower extremities was defined using the International Classification of Diseases, 9th Revision [20] code 4439. This code is described as peripheral vascular disease unspecified, intermittent claudication not otherwise specified (NOS). Peripheral: angiopathy NOS, vascular disease NOS, Spasm of artery. Excluded are atherosclerosis of the arteries of the extremities and spasm of cerebral artery.

The ICD code does not provide information on severity. We assume that patients with moderate-severe claudication, ischemic rest pain and ulceration or gangrene (Fontaine stage IIb, III and IV, respectively) are hospitalized as these patients have an indication for surgical interventions [3]

### Study population

All hospital admissions for PAD in the lower extremities between January 1^st ^and December 31^st^, 1997 and January 1^st ^and December 31^st^, 2000, were selected from the national hospital discharge registry (which was previously linked with PR). There were 7,161 hospital admissions. Selection of the first admission for an individual of all subsequent admissions of a person occurring 1997 and 2000 yielded a total of 5,886 patients with PAD in the lower extremities. Subsequently, information was collected on hospital admission that may have occurred previously (1995–1997(data earlier than 1995 not available since linkage with PR is only possible from 1995) and 1995–2000, respectively) for the same condition. Those with a previous admission for peripheral arterial disease were excluded (n = 1,728). This resulted in a cohort consisting of 4,158 patients with a first hospitalization for PAD in the lower extremities in 1997 or 2000 in the Netherlands.

### Co-morbidity

The presence of co-morbidity (cardiovascular disease (ICD-9-CM codes 390–459) or diabetes mellitus (DM) (ICD-9-CM code 250)) was determined on the basis of the discharge diagnosis of previous hospital admissions or on the basis of a secondary diagnosis at the time of the index admission. No information in the registry was available on the severity of disease, risk factor (hypertension, smoking) or medication use.

### Follow-up

Information on mortality was obtained by linkage of the cohort with national cause of death register. Linkage of the PR (which was linked previously to the HDR cohort) with the cause of death register was performed using an unique identification key and therefore was nearly complete. Patients were censored if they migrated out of the Netherlands or if their linkage key was not unique anymore during follow-up. Causes of death were coded using the tenth revision of the International Classification of Disease (ICD-10) [21].

### Data analysis

Survival time was calculated as the time from the initial admission date for IC in 1997 or 2000 to the date of death from any cause or to the date that a patient was censored, whichever came first. The crude short-term (28 day), 1 year and long-term (5-year) mortality were computed by age and gender according to the actuarial life table method and expressed as percentages. The mortality rate in men was compared to mortality rate in women by calculating relative risks (with 95% CI). Cox regression models were used to study differences between men and women in their risk of death. Mortality at 28-days, 1-year and 5-years was examined. For each period, a model was fitted to adjust for for age and co-morbidity (previous admissions for cardiovascular disease or DM). Data were analyzed with SPSS software, version 14.0 (SPSS Inc, Chicago, Illinois, USA). All analyzes were performed in agreement with privacy legislation in the Netherlands [22].

## Results

A total of 4,158 patients with a first hospital admission for a PAD in the lower extremities in 1997 or 2000 were identified. General characteristics are provided in Table 1. Two percent, 10% and 31% of all men and 4%, 10% and 27% of all women died within 28 days, 1-year and 5-years, respectively.

**Table 1:** Characteristics of patients with a first hospitalization for PAD in the lower extremities in 1997 or 2000.

	Men	Women	Total
Number of patients	2,539	1,619	4,158

Age at admission (years)			
Mean	66.1	66.9	66.4
Standard deviation	11.6	13.9	12.6

Previous hospital admission in 1995–1997, 1995–2000 (%)			
cardiovascular disease	28.9*	25.0	27.3
- ischemic heart disease	13.6*	8.6	11.7
- acute myocardial infarction	3.7	3.2	3.5
- congestive heart failure	3.9	3.5	3.8
- stroke	4.6*	3.3	4.1
- other cardiovascular disease	14.0	15.2	14.5
diabetes mellitus	10.8	13.3*	11.8

Type of hospital (%)			
- academic	11.2*	7.8	9.9

Length of stay (days)			
Mean	4	5	4

Origin (%)			
-native	91.5	89.5	90.7

### Cause of death

Cardiovascular diseases were the most frequent cause of death at 28-days, 1-year and 5-years (Table 2). The contribution of cardiovascular diseases as cause of death decreased over time while the contribution of cancer as a cause of death increased.

**Table 2:** Causes of death at 28-days, 1-year and 5-years of patients with a first hospitalization for PAD in the lower extremities in the Netherlands.

Cause of death	28 days		1-year		5-years	
	Men	Women	Men	Women	Men	Women
	(n = 60)	(n = 57)	(n = 262)	(n = 168)	(n = 786)	(n = 443)
Cardiovascular diseases	70.0	68.4	55.0	61.9	50.3	53.7
- ischemic heart disease	23.3	12.3	14.5	15.5	17.4	14.7
- AMI	11.7	8.8	7.6	11.3	10.3	10.4
- congestive heart failure	5.0	7.0	5.7	6.5	4.8	4.7
- stroke	1.7	7.0	9.2	8.3	8.9	7.9
- peripheral arterial diseases	33.3	35.1	19.8	22.6	12.1	15.3
- other cardiovascular diseases	6.7	7.0	5.7	8.9	7.0	11.1
Cancer	5.0	1.8	13.7*	6.5	18.1	12.6
- lung cancer	3.3	0.0	5.7	2.4	6.9	3.8
Diseases of respiratory system	11.7	7.0	9.5	6.5	9.9	8.1
- COPD	5.0	5.3	5.0	3.6	5.2	3.8
Complications from DM	8.3	12.3	8.8	11.9	7.3	10.8
Other	5.0	10.5	13.0	13.2	14.4	14.8

* Age-adjusted gender difference significant

Values are number of deaths expressed as a percentage of total number of deaths

AMI: acute myocardial infarction, COPD: chronic obstructive respiratory disease, DM: diabetes mellitus

### 28-day mortality

Short term mortality risk increased with age in men and women (from 2.3% in men between 65–69 years to 18.4% in men older than 85 years and from 2.3% in women between 50–54 years to 18.8% in women older than 85 years) (Table 3). The crude overall mortality was lower for men (relative risk 0.67; 95% CI 0.47 to 0.96). After adjustment for potential confounding factors no differences between men and women in mortality were observed (Table 4).

**Table 3:** Mortality risk at 28 days, 1 year and 5 years after a first hospital admission (1997 or 2000) for PAD in the lower extremities in the Netherlands, by age and gender.

				Men		Women		RR (95% CI) for Men vs Women
					
	No. of men	No. of women	Age	No. of Deaths	Percentage Deaths	No. of Deaths	Percentage Deaths	
28-day mortality	109	116	< 45	-	-	-	-	-
	133	95	45–49	-	-	-	-	-
	236	128	50–54	-	-	3	2.3	-
	263	116	55–59	-	-	-	-	-
	322	162	60–64	-	-	-	-	-
	444	215	65–69	10	2.3	4	1.9	1.21 (0.38–3.82)
	425	285	70–74	6	1.4	3	1.1	1.34 (0.34–5.32)
	363	242	75–79	15	4.1	11	4.5	0.91 (0.42–1.95)
	168	143	80–84	13	7.7	11	7.7	1.0 (0.47–2.18)
	76	117	85+	14	18.4	22	18.8	0.98 (0.54–1.79)
	2,539	1,619	all ages	60	2.4	57	3.5	

1-year mortality	109	116	< 45	2	1.8	-	-	-
	133	95	45–49	3	2.3	-	-	-
	236	128	50–54	7	3.0	5	3.9	0.75 (0.25–2.34)
	263	116	55–59	7	2.7	3	2.6	1.03 (0.27–3.91)
	322	162	60–64	22	6.8	9	5.6	1.23 (0.58–2.61)
	444	215	65–69	36	8.1	14	6.5	1.25 (0.69–2.26)
	425	285	70–74	50	11.8	23	8.1	1.46 (0.91–2.33)
	363	242	75–79	55	15.2	31	12.8	1.18 (0.79–1.78)
	168	143	80–84	44	26.2	33	23.1	1.13 (0.77–1.68)
	76	117	85+	36	47.7	48	41.0	1.15 (0.84–1.59)
	2,539	1,619	all ages	262	10.3	168	10.4	

5-year mortality	109	116	< 45	9	8.3	2	1.7	4.79 (1.06–21.7)
	133	95	45–49	10	7.5	4	4.2	1.79 (0.58–5.52)
	236	128	50–54	21	8.9	11	8.6	1.04 (0.52–2.08)
	263	116	55–59	37	14.1	7	6.0	2.33 (1.07–5.07)
	322	162	60–64	73	22.7	31	19.1	1.18 (0.81–1.72)
	444	215	65–69	136	30.6	53	24.7	1.24 (0.95–1.63)
	425	285	70–74	157	36.9	74	26.0	1.42 (1.12–1.79)
	363	242	75–79	171	47.1	100	41.3	1.14 (0.95–1.37)
	168	143	80–84	108	64.3	74	51.7	1.24 (1.02–1.51)
	76	117	85+	64	84.2	87	74.4	1.13.(0.98–1.31)
	2,539	1,619	all ages	786	31.0	443	27.4	

- Data about 6 patients (male <65 or women <50 and from 55 to 64 year) were not available as a consequence of privacy regulation

**Table 4:** Gender differences in short- and long-term mortality after a first hospital admission (1997 or 2000) for PAD in the lower extremities in the Netherlands

	PAD in the lower extremities
	
	HR (95% CI)*
Crude	
28 days	0.67 (0.47–0.96)
1 year	0.90 (0.81–1.20)
5 years	1.15 (1.03–1.29)
	
Model I	
28 days	0.85 (0.59–1.24)
1 year	1.17 (0.97–1.43)
5 years	1.36 (1.21–1.53)

Hazard ratio (95% Confidence interval) men vs women.

Model I: adjusted for age, co-morbidity (previous admissions for cardiovascular disease or diabetes mellitus)

### One-year mortality

Mortality increased with age in men and women (from 1.8% in men younger than 45 years to 47.7% in men older than 85 years and from 3.9% in women between 50–54 years to 41.0% in women older than 85 years). A higher mortality in men compared with women was found across all ages above 55 years, however differences were not statistically significant (Table 3). The mortality risks at 1-year were similar for men and women (Table 4).

### Five-year mortality

Mortality risk at 5-year was 8.3% for men younger than 45 years, 84.2% for men older than 85 years, 1.7% for women younger than 45 years and 74.4% for women older than 85 year (Table 4). The crude overall mortality was higher for men (relative risk 1.31;95% CI 1.03 to 1.25) which persisted after adjustment for potential confounding factors (Table 4).

## Discussion

This study provides estimates of absolute short- and long-term risk of death after an initial hospital admission for PAD in the lower extremities. Our findings demonstrate that, although short-term mortality risk after initial admission for PAD in the lower extremities is relatively low, 5-year mortality risk is high, especially in men.

One-year mortality rates were higher in the present study than those reported in intervention studies evaluating the effect of drug treatment for IC [23-25]. In these trials, mortality risks (around 1.2% after 1 year) were much lower than the mortality risk in our study population (mortality risk 10.4% after 1 year). This likely reflects exclusion of the sickest patients from participation in trials [26]. Gender stratified 1-year and 5-years mortality risks (6% and 38% in men and 4% and 20% in women, respectively) that were reported for patients first seen with IC in hospital [5] were also somewhat lower in comparison with our findings, except for 5-years mortality in men. Much higher gender stratified 1-year and 5-years mortality risks (22% and 67% in men and 17% and 62% in women, respectively) have been reported for patients with CLI [27]. However, comparison between these studies and our study is difficult, as the baseline clinical characteristics of the patients in the present study are mostly unknown.

Whereas studies reporting stratified (for age and/or gender) mortality risks for patients with PAD in lower extremities are scarce, many studies reported mortality risks for PAD patients stratified by ankle-brachial index (ABI) [14,16,28]. These studies indicate that a low ABI is associated with high mortality risk. Five-year mortality risks of 16% and 34% have been reported for patients with ABI 0.71–0.9 and <0.7, respectively [15] and 34% and 56% for patients with ABI 0.4–0.85 and ABI <0.4 [28]. The getABI study [29] reported higher risk of death in patients with peripheral disease (low ABI) compared with those without (hazard ratio 2.3), suggesting that ABI may be a good risk predictor beyond conventional risk predictors [30].

**Figure 1:**
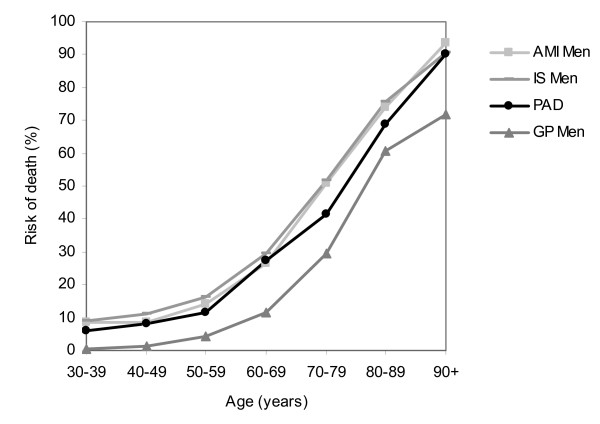
Mortality risk at 5 years* after a first hospital admission for PAD in the lower extremities, acute myocardial infarction and ischemic stroke, by age. Men * Mortality risks AMI, IS and GP extracted from other data [18,31,47] AMI: Acute myocardial Infarction, IS: Ischemic stroke, PAD: Peripheral arterial disease in the lower extremities, GP: General population

**Figure 2:**
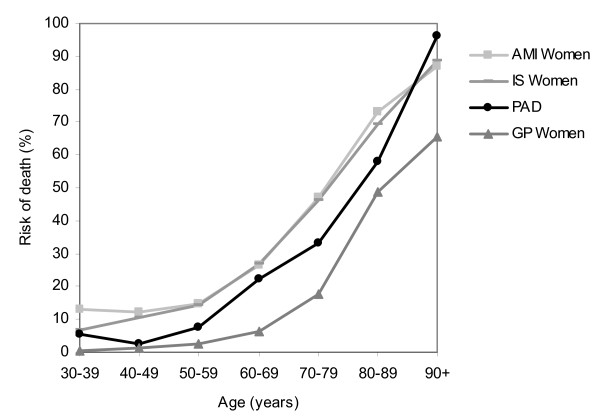
Mortality risk at 5 years* after a first hospital admission for PAD in the lower extremities, acute myocardial infarction and ischemic stroke, by age. Women * Mortality risks AMI, IS and GP extracted from other data [18,31,47] AMI: Acute myocardial Infarction, IS: Ischemic stroke, PAD: Peripheral arterial disease in the lower extremities, GP: General population

The long-term risk of death of patients with a first hospitalization for PAD in the lower extremities is high compared to risk of death in the general population (Figure 1 and Figure 2). Furthermore, comparison with survival after admission for other atherosclerotic diseases such as acute myocardial infarction and ischemic stroke showed similar mortality risks (Figure 1 and Figure 2) [13,18,31]. In contrast, awareness of the severity of PAD in the lower extremities is low as in general population the familiarity with this disease is significantly lower than that for any other cardiovascular disease. A recent study showed that 26% of the public was familiar with the disorder and of those only 14% was aware that the disease could lead to death [32]. Furthermore, PAD patients tend to be undertreated compared to patients with other manifestations of atherosclerosis and it seems that cardiovascular risk factor management for prevention in PAD patients is very modest [33,34].

Strength of our study is the large size of the cohort obtained from usual care with a large age range and information on both men and women. Even though the validity of national registries has been questioned, several studies have shown that for the Netherlands, the validity is adequate [35,36]. Furthermore a high validity of the linkage between them has been demonstrated [36,37].

We provided detailed information on age- and gender-specific risks, while a large number of publications, including institutional and population-based studies, simply present overall mortality risks of groups of patients without the presentation of the information by age and gender, despite the evidence that mortality depends on age and gender.

Besides age and gender, there are many other factors that may contribute to the prognosis of PAD patients. The stage of PAD, presence of traditional cardiovascular risk factors (e.g. smoking) [38], physical inactivity [39], systemic inflammation [40], impaired renal function [41], hypercholesterolemia, statin use and ABI lower than 0.60 [14] have shown to influence the prognosis of PAD patients. Unfortunately, no information in the registry was available on these factors and therefore there cannot be adjusted for these factors in the analysis of the data. On the other hand, our primary goal was to provide absolute mortality risks stratified by age and gender as absolute risks are easier to interpret and they provide a clear presentation of the size of the burden of disease in these patients, which may be helpfull to increase awareness, as it makes the problem as clear as possible.

Further aspects of our study that need critical consideration are 1.) the study population is restricted to patients with a first admission for PAD in the lower extremities. Non-hospitalized patients were not included, while those admitted for PAD in the lower extremities are most likely more severe patients compared to those not admitted and may therefore have a higher mortality risk as compared to the entire group of patients with PAD in the lower extremities. 2.) In our data we found a higher percentage of deaths after initial admission for PAD in the lower extremities due to lung cancer and respiratory diseases in men as compared to women. This might be viewed as a higher smoking prevalence among men. Smoking is one of the major risk factors for the development of PAD in the lower extremities [42-44], the prevalence is higher among men [45] and smoking reduces survival in PAD patients [46]. Smoking behaviour might affect our results through overestimation of the gender differences in mortality risk. However, exclusion of the deaths caused by lungcancer and respiratory diseases did not change the gender differences in mortality risk (data not shown). 3.) The limitation of previous admissions to maximal 6 years, might have led to that some "first" patients with PAD in lower extremities were actually recurrent patients with PAD in the lower extremities. These may be more severe patients and on the premise that severity is associated with a higher mortality risk, possibly leading to some overestimation of the mortality risk. The extent of which is difficult to quantify.

## Conclusion

Our study provides nationwide estimates of short- and long-term risk of death after an initial hospital admission for PAD in the lower extremities. Our findings demonstrate that, 5-years mortality risk is high, especially in men and comparable to that of patients admitted for acute myocardial infarction or ischemic stroke. Though, in general population the awareness of the severity of PAD in the lower extremities is significantly lower than that for any other cardiovascular disease and it seems that cardiovascular risk factor management for prevention in PAD patients is very modest.

## Competing interests

The authors declare that they have no competing interests.

## Authors' contributions

IV performed the statistical analysis and drafted the manuscript. GB drafted the manuscript. JR participated in the design of this study and commented the draft. AB participated in the design of this study and its coordination. FM conceived of the study and commented the draft. DG conceived of the study and commented the draft. MB conceived of the study, and participated in its design and coordination. All authors read and approved the final manuscript.

## Pre-publication history

The pre-publication history for this paper can be accessed here:


